# Supplementary material for: The Stronger Downregulation of in vitro and in vivo Innate Antiviral Responses by a Very Virulent Strain of Infectious Bursal Disease Virus (IBDV), Compared to a Classical Strain, Is Mediated, in Part, by the VP4 Protein
Source: Front Cell Infect Microbiol. 2020 Jun 9;10:315. doi: 10.3389/fcimb.2020.00315 (PMC7296162; doi:10.3389/fcimb.2020.00315)
Supplement: Supplementary file 1 [file Data_Sheet_1.PDF]

Supplementary Table 1: Primers used in this study for qPCR.

Name	Sequence (5'-3')		
	Forward	Reverse	Probe or SYBR Green
**IBDV**	GAGGTGGCCGACCTCAACT	GCCCGGATTATGTCTTTGAAG	Probe: FAM-CCCCTGAAGATTGCAG GAGCATT-TAMRA
**IL-6**	AACATGCGTCAGCTCCTGAAT	TCTGCTAGGAACTTCT CCATTGAA	SYBR Green
**IL-1β**	GCTCTACATGTCGTGTGTGATGAG	TGTCGATGTCCCGCATGA	SYBR Green
**IL-8**	GCCCTCCTCCTGGTTTCAG	TGGCACCGCAGCTCATT	SYBR Green
**Mx1**	CACACCCAACTGTCAGCGAT	ATGTCCGAAACTCTCTGCGG	SYBR Green
**IFNα**	CCACCGCTACACCCAGCACC	ATGGTGAGGTGAAGGTTGCGA	SYBR Green
**IFNβ**	CAGTCTCCAGGGATGCACAG	GAGAAGGTGGTGGTGAGAGC	SYBR Green
**RPLP0**	TTGGGCATCACCACAAAGATT	CCCACTTTGTCTCCGGTCTTAA	SYBR Green
**TBP**	CTTCGTGCCCGAAATGCT	GCGCAGTAGTACGTGGTTCTCTT	SYBR Green

(E) indicates the bird should be euthanized immediately.

Appearance:
This is done by accumulative scoring.

Normal appearance	0
Pale comb / wattles	+1
Fluffed out feathers	+1
Eyes half shut	+1
Drooping wings / hunched / hangs head	+1
Laboured breathing	+1

Behaviour without provocation:
Select the score suited to the individual being assessed.

Normal posture	0
Crowds with group, but alert and social interactions with other birds evident, efforts made to seek water / feed in an independent manner.	1
Crowds within group, but lacking signs of normal social interaction / looking depressed, no efforts made to seek water / feed in an independent manner	2
Stands alone, depressed and no normal social interaction or independent action	3
Laid down alone, depressed and no normal social interaction or independent action	4
Collapsed with clear signs of severe distress	5 (E)

Provoked behaviour:
Select the score suited to the individual being assessed.

Normal (escapes capture by running)	0
Normal posture and attempts to avoid capture (escape attempted by walking)	1
Weak response to capture	2
No attempt to avoid capture but struggles when picked up	3
No attempt to avoid capture and lethargic when picked up	4
Fails to move when provoked	5 (E)

Handling:
This is done by accumulative scoring.

Feels normal	0
Fails to gain weight	+1
Evidence of weight loss	+1
Crop filled with water	+1
Soiled vent with classic white faecal material	+1

- Infected birds must be checked and weighed twice daily from the onset of clinical signs until after the infection has cleared and no more clinical signs are present. When no clinical signs are present, the birds can be checked once daily.
- Birds scoring 8-11 are classed as having moderate disease and must be checked every 8 hours
- Birds scoring 12-17 are classed as having have severe disease and must be checked every 3 hours
- Birds scoring 17 or E must be humanely culled.
- Birds scoring 12 for 3 days must be humanely culled.

Supplementary Figure 1. Clinical Scoring system used in *in vivo* studies. Birds were checked at least twice daily following inoculation by two independent observers and the clinical signs scored. Birds were humanely culled when their humane end-points were reached (a clinical score of 11 in this study).

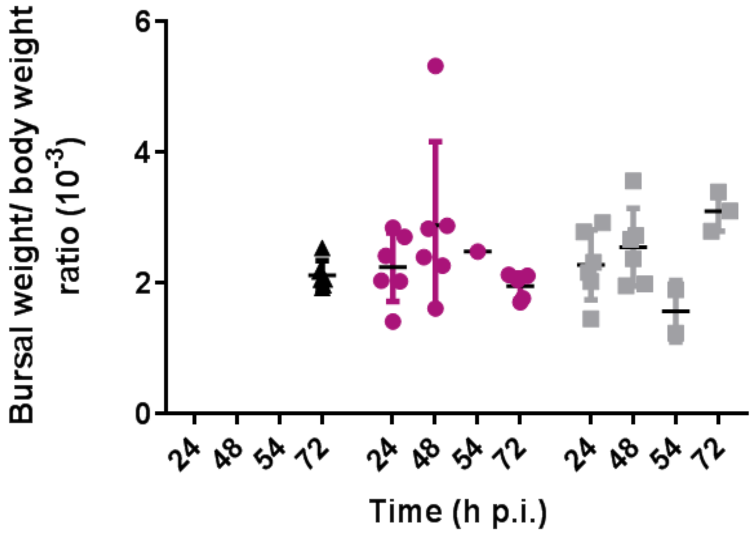

Supplementary Figure 2: No significant difference was observed between the Bursa of Fabricius: body weight (BF:BW) ratios of mock, F52/70- or UK661-inoculated birds. Six birds per group were humanely culled at 24 and 48 hpi. One bird infected with F52/70 and three birds infected with UK661 were humanely culled at 54 hpi due to reaching their humane end points. The remaining infected birds were humanely culled at 72 hpi. 6 mock-infected birds were also humanely culled at 72 hpi. Birds were weighed and the bursa of Fabricius was removed at necropsy and weighed. The BF:BW ratio was determined and plotted for each bird. Horizontal lines represent the mean and error bars represent the standard error of the mean (SEM).

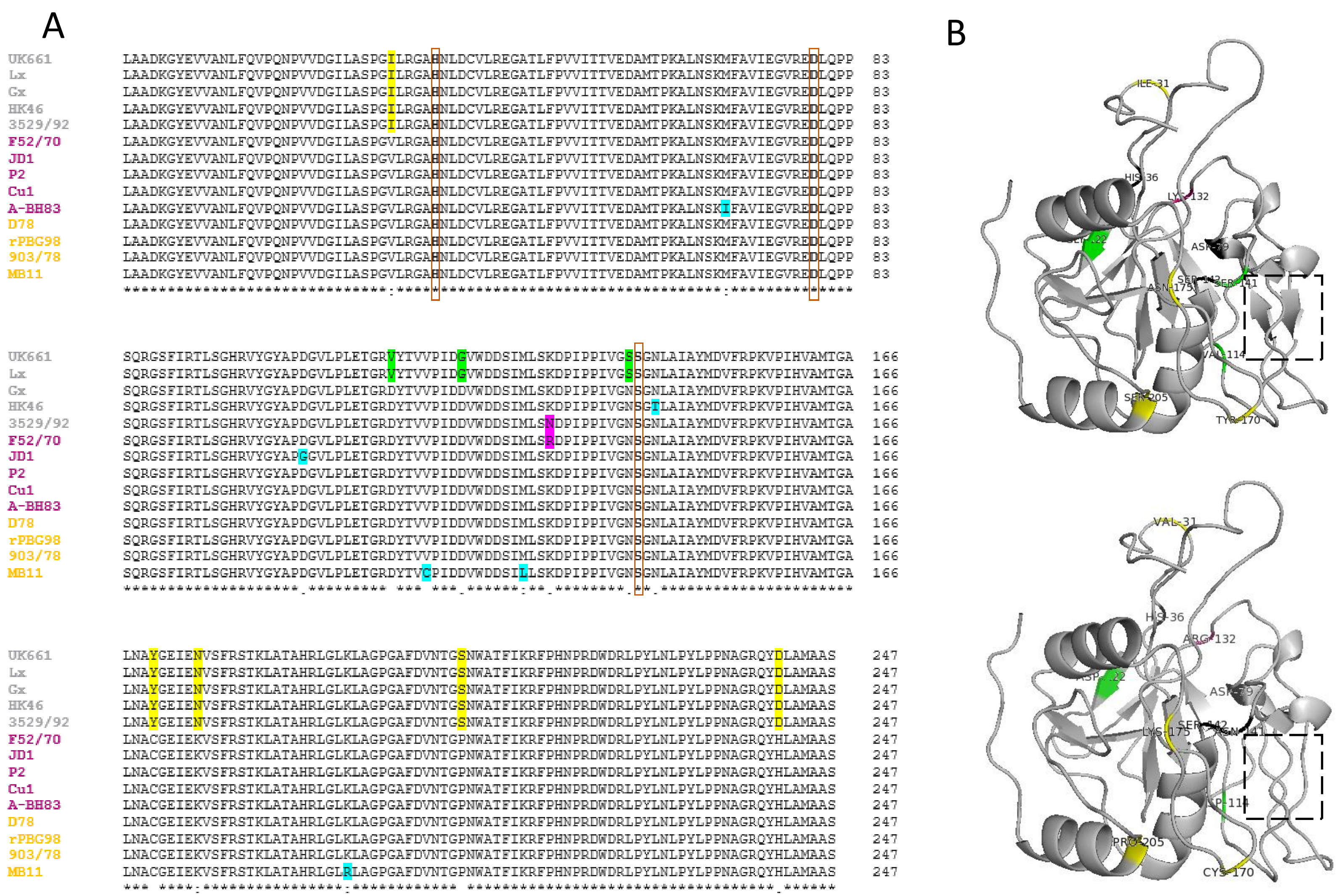
A
UK661 LAADKGYEVVANLFQVPQNPVVDGILASPGILRGAHNLDCVLREGATLFPVVITTVEDAMTPKALNSKMFAVIEGVREDLQPP 83
Lx LAADKGYEVVANLFQVPQNPVVDGILASPGILRGAHNLDCVLREGATLFPVVITTVEDAMTPKALNSKMFAVIEGVREDLQPP 83
Gx LAADKGYEVVANLFQVPQNPVVDGILASPGILRGAHNLDCVLREGATLFPVVITTVEDAMTPKALNSKMFAVIEGVREDLQPP 83
HK46 LAADKGYEVVANLFQVPQNPVVDGILASPGILRGAHNLDCVLREGATLFPVVITTVEDAMTPKALNSKMFAVIEGVREDLQPP 83
3529/92 LAADKGYEVVANLFQVPQNPVVDGILASPGILRGAHNLDCVLREGATLFPVVITTVEDAMTPKALNSKMFAVIEGVREDLQPP 83
F52/70 LAADKGYEVVANLFQVPQNPVVDGILASPGVLRGAHNLDCVLREGATLFPVVITTVEDAMTPKALNSKMFAVIEGVREDLQPP 83
JD1 LAADKGYEVVANLFQVPQNPVVDGILASPGVLRGAHNLDCVLREGATLFPVVITTVEDAMTPKALNSKMFAVIEGVREDLQPP 83
P2 LAADKGYEVVANLFQVPQNPVVDGILASPGVLRGAHNLDCVLREGATLFPVVITTVEDAMTPKALNSKMFAVIEGVREDLQPP 83
Cu1 LAADKGYEVVANLFQVPQNPVVDGILASPGVLRGAHNLDCVLREGATLFPVVITTVEDAMTPKALNSKMFAVIEGVREDLQPP 83
A-BH83 LAADKGYEVVANLFQVPQNPVVDGILASPGVLRGAHNLDCVLREGATLFPVVITTVEDAMTPKALNSKIFAVIEGVREDLQPP 83
D78 LAADKGYEVVANLFQVPQNPVVDGILASPGVLRGAHNLDCVLREGATLFPVVITTVEDAMTPKALNSKMFAVIEGVREDLQPP 83
rPBG98 LAADKGYEVVANLFQVPQNPVVDGILASPGVLRGAHNLDCVLREGATLFPVVITTVEDAMTPKALNSKMFAVIEGVREDLQPP 83
903/78 LAADKGYEVVANLFQVPQNPVVDGILASPGVLRGAHNLDCVLREGATLFPVVITTVEDAMTPKALNSKMFAVIEGVREDLQPP 83
MB11 LAADKGYEVVANLFQVPQNPVVDGILASPGVLRGAHNLDCVLREGATLFPVVITTVEDAMTPKALNSKMFAVIEGVREDLQPP 83
UK661 SQRGSFIRTLSGHRVYGYAPDGVLPLETGRVYTVVPIDGVWDDSIMLSKDPIPPIVGSSGNLAIAYMDVFRPKVPIHVAMTGA 166
Lx SQRGSFIRTLSGHRVYGYAPDGVLPLETGRVYTVVPIDGVWDDSIMLSKDPIPPIVGSSGNLAIAYMDVFRPKVPIHVAMTGA 166
Gx SQRGSFIRTLSGHRVYGYAPDGVLPLETGRDYTVVPIDDVWDDSIMLSKDPIPPIVGNSGNLAIAYMDVFRPKVPIHVAMTGA 166
HK46 SQRGSFIRTLSGHRVYGYAPDGVLPLETGRDYTVVPIDDVWDDSIMLSKDPIPPIVGNSGTLAIAYMDVFRPKVPIHVAMTGA 166
3529/92 SQRGSFIRTLSGHRVYGYAPDGVLPLETGRDYTVVPIDDVWDDSIMLSNDPIPPIVGNSGNLAIAYMDVFRPKVPIHVAMTGA 166
F52/70 SQRGSFIRTLSGHRVYGYAPDGVLPLETGRDYTVVPIDDVWDDSIMLSRDPIPPIVGNSGNLAIAYMDVFRPKVPIHVAMTGA 166
JD1 SQRGSFIRTLSGHRVYGYAPGGVLPLETGRDYTVVPIDDVWDDSIMLSKDPIPPIVGNSGNLAIAYMDVFRPKVPIHVAMTGA 166
P2 SQRGSFIRTLSGHRVYGYAPDGVLPLETGRDYTVVPIDDVWDDSIMLSKDPIPPIVGNSGNLAIAYMDVFRPKVPIHVAMTGA 166
Cu1 SQRGSFIRTLSGHRVYGYAPDGVLPLETGRDYTVVPIDDVWDDSIMLSKDPIPPIVGNSGNLAIAYMDVFRPKVPIHVAMTGA 166
A-BH83 SQRGSFIRTLSGHRVYGYAPDGVLPLETGRDYTVVPIDDVWDDSIMLSKDPIPPIVGNSGNLAIAYMDVFRPKVPIHVAMTGA 166
D78 SQRGSFIRTLSGHRVYGYAPDGVLPLETGRDYTVVPIDDVWDDSIMLSKDPIPPIVGNSGNLAIAYMDVFRPKVPIHVAMTGA 166
rPBG98 SQRGSFIRTLSGHRVYGYAPDGVLPLETGRDYTVVPIDDVWDDSIMLSKDPIPPIVGNSGNLAIAYMDVFRPKVPIHVAMTGA 166
903/78 SQRGSFIRTLSGHRVYGYAPDGVLPLETGRDYTVVPIDDVWDDSIMLSKDPIPPIVGNSGNLAIAYMDVFRPKVPIHVAMTGA 166
MB11 SQRGSFIRTLSGHRVYGYAPDGVLPLETGRDYTVCPIDDVWDDSILLSKDPIPPIVGNSGNLAIAYMDVFRPKVPIHVAMTGA 166
UK661 LNAYGEIENVSFRSTKLATAHRLGLKLAGPGAFDVNTGSNWATFIKRFPHNPRDWDRLPYLNLPYLPPNAGRQYDLAMAAS 247
Lx LNAYGEIENVSFRSTKLATAHRLGLKLAGPGAFDVNTGSNWATFIKRFPHNPRDWDRLPYLNLPYLPPNAGRQYDLAMAAS 247
Gx LNAYGEIENVSFRSTKLATAHRLGLKLAGPGAFDVNTGSNWATFIKRFPHNPRDWDRLPYLNLPYLPPNAGRQYDLAMAAS 247
HK46 LNAYGEIENVSFRSTKLATAHRLGLKLAGPGAFDVNTGSNWATFIKRFPHNPRDWDRLPYLNLPYLPPNAGRQYDLAMAAS 247
3529/92 LNAYGEIENVSFRSTKLATAHRLGLKLAGPGAFDVNTGSNWATFIKRFPHNPRDWDRLPYLNLPYLPPNAGRQYDLAMAAS 247
F52/70 LNACGEIEKVSFRSTKLATAHRLGLKLAGPGAFDVNTGPNWATFIKRFPHNPRDWDRLPYLNLPYLPPNAGRQYHLAMAAS 247
JD1 LNACGEIEKVSFRSTKLATAHRLGLKLAGPGAFDVNTGPNWATFIKRFPHNPRDWDRLPYLNLPYLPPNAGRQYHLAMAAS 247
P2 LNACGEIEKVSFRSTKLATAHRLGLKLAGPGAFDVNTGPNWATFIKRFPHNPRDWDRLPYLNLPYLPPNAGRQYHLAMAAS 247
Cu1 LNACGEIEKVSFRSTKLATAHRLGLKLAGPGAFDVNTGPNWATFIKRFPHNPRDWDRLPYLNLPYLPPNAGRQYHLAMAAS 247
A-BH83 LNACGEIEKVSFRSTKLATAHRLGLKLAGPGAFDVNTGPNWATFIKRFPHNPRDWDRLPYLNLPYLPPNAGRQYHLAMAAS 247
D78 LNACGEIEKVSFRSTKLATAHRLGLKLAGPGAFDVNTGPNWATFIKRFPHNPRDWDRLPYLNLPYLPPNAGRQYHLAMAAS 247
rPBG98 LNACGEIEKVSFRSTKLATAHRLGLKLAGPGAFDVNTGPNWATFIKRFPHNPRDWDRLPYLNLPYLPPNAGRQYHLAMAAS 247
903/78 LNACGEIEKVSFRSTKLATAHRLGLKLAGPGAFDVNTGPNWATFIKRFPHNPRDWDRLPYLNLPYLPPNAGRQYHLAMAAS 247
MB11 LNACGEIEKVSFRSTKLATAHRLGLRLAGPGAFDVNTGPNWATFIKRFPHNPRDWDRLPYLNLPYLPPNAGRQYHLAMAAS 247
B
ILE-31
HIS-36
LYS-132
ASP-79
SER-142
ASN-175
SER-141
VAL-114
SER-205
TYR-170
VAL-31
HIS-36
ARG-132
ASP-79
LYS-175
SER-142
ASN-141
PRO-205
CYS-170

Supplementary Figure 3. There are nine amino acids different between the UK661 and the F52/70 VP4 proteins that cause differences to the predicted secondary structure of the molecule. The amino acid sequences of five very virulent (grey), six classical (pink) and four cell-adapted (orange) IBDV strains were aligned by Clustal (GenomeNet) (A). Amino acids highlighted in yellow are exclusive to vvIBDV strains and those in pink are specific to F52/70 compared to all other strains. Residues highlighted in green are found in some but not all vvIBDV strains, and residues highlighted in blue indicate other amino acid differences in the VP4 sequences. The boxed regions are the catalytic triad responsible for the protease function of VP4. The Predicted UK661 (top) and F52/70 (bottom) VP4 structures based on the Yellowtail Ascites Virus VP4 (template 4izk.2.A) modelled using PyMol (B). Mutations between F52/70 and UK661 are labelled in the same colours as indicated in (A) and black sites indicate the location of the catalytic triad responsible for protease function. The dashed- boxed region shows examples of altered secondary structure, with alpha helices present in the UK661 VP4 molecule that are not seen in the F52/70 VP4 molecule.
